# Associations between symptom severity and well-being among Thai patients with schizophrenia: a cross-sectional analytical study

**DOI:** 10.1186/s12888-021-03358-0

**Published:** 2021-07-12

**Authors:** Teerapat Teetharatkul, Arnont Vitayanont, Tippawan Liabsuetrakul, Warut Aunjitsakul

**Affiliations:** 1 Department of Psychiatry, Faculty of Medicine, Prince of Songkla University, Hat Yai, Songkhla 90110 Thailand; 2 Epidemiology Unit, Faculty of Medicine, Prince of Songkla University, Songkhla, 90110 Thailand

**Keywords:** Affective symptoms, Antipsychotic agents, Happiness, Psychotic disorders, Quality of life

## Abstract

**Background:**

Severity of symptoms in patients with schizophrenia is a determinant of patient’s well-being, but evidence in low- and middle-income countries is limited. We aimed to measure the symptom severity using objective measurements, the Brief Psychiatric Rating Scale (BPRS) and Clinical Global Impression-Severity scale (CGI-S), and their associations with well-being in patients with schizophrenia.

**Methods:**

Patients with schizophrenia aged ≥18 years, without active psychosis including no history of hospitalization within the last 6 months, were included. Symptom severity was measured by the clinicians using BPRS and CGI-S. The patients’ well-being was assessed by self-report using the Subjective Well-being under Neuroleptic treatment scale (SWN) as continuous and binary outcomes (categorized into adequate or poor well-being). Correlations between symptom severity (BPRS and CGI-S scores) and well-being (SWN score) were analyzed using Pearson’s correlation. Association between well-being status and BPRS was analyzed using multivariate logistic regression.

**Results:**

Of 150 patients, BPRS and CGI-S were inversely correlated with SWN score (*r* = − 0.47; *p* < 0.001 and − 0.21; *p* < 0.01, respectively). BPRS Affect domain had the highest correlation with SWN (*r* = − 0.51, p < 0.001). In multivariate logistic regression, BPRS score and being unemployed were associated with poor well-being status (adjusted OR 1.08; 95%CI 1.02–1.14; *p* = 0.006, and 4.01; 95%CI 1.38–11.7; *p* = 0.011, respectively).

**Conclusion:**

Inverse relationships between symptom severity and well-being score were found. Higher BPRS Affect domain was significantly associated with lower patients’ well-being. The use of BPRS tool into routine clinical practice could serve as an adjunct to physician’s clinical evaluation of patients’ symptoms and may help improve patient’s well-being. Further research on negative symptoms associated with well-being is required.

## Background

Physicians generally render their judgments for clinical management. Although this is a common practice, there are common fallibilities (e.g. the intellect of clinicians, and lack of error checking) of the judgments that may affect the reliability of the physician’s assessment [1]. It was suggested that the approach to reduce physician error is to provide more double-checking and awareness of uncommon symptoms [1]. Therefore, regarding the improvement of clinical assessment, it is important to utilize measurement of symptom severity together with using physician’s assessment.

Although people with schizophrenia in remission do not present clinically active psychosis, they seem not to recover over time despite more effective management (e.g. deinstitutionalization, antipsychotic medications, psychosocial interventions, and early psychosis services) [2]. Through the course of illness, they experience heterogeneous impairments including cognition deficit, [3–5] poor physical health [6, 7] and functional disabilities [5]. Furthermore, they generally suffer from psychiatric comorbidities such as depression and anxiety [8]. This results in a significant negative impact on their subjective well-being and quality of life [9–11].

Several studies have focused on investigating the symptom severity associated with patients’ well-being in schizophrenia, [12–16] but evidence from low- and middle-income countries, like Thailand, is still limited. We hoped that the use of additional tools rather than physician’s assessment alone could help assess symptom severity by identifying a specific symptom that the physician should focus on for targeted treatment in improvement of patients’ well-being. This study aimed to measure symptom severity using the clinical measurements, and their association with patients’ well-being. We hypothesized that an inverse relationship would be found between symptom severity and well-being.

## Methods

### Study design

We conducted a cross-sectional study in outpatients with schizophrenia at the Department of Psychiatry, Prince of Songkla University (PSU), Thailand. This study was approved by the Ethics Committee of the Faculty of Medicine, PSU (REC: 60–197–03-1), under the ethical principles of the Declaration of Helsinki.

### Participants

Outpatients with schizophrenia (diagnosed by the 10th revision of the International Statistical Classification of Diseases and Related Health Problems; ICD-10), aged from 18-year-old, with no acute psychotic symptoms and without a history of hospitalization within 6 months were included. Participants were excluded if they were unable to communicate in Thai fluently, unable to read and write in Thai language, had a serious or unstable physical illness, or were substance-dependent.

### Measurement tools

We collected socio-demographic data (e.g. sex, age, highest education, employment, monthly income) and medical history (e.g. duration of illness, history of hospitalization, antipsychotic treatments and side effects). Antipsychotic treatments were recorded including types (typical or first-generation, and atypical or second-generation), routes (oral and intramuscular) and a dose of antipsychotic drug. The antipsychotic daily dose was calculated as chlorpromazine (CPZ) - equivalent dose, for example, 1 mg haloperidol or risperidone equivalent to 100 mg CPZ [17].

#### Symptom severity measurements

The primary measure of interest was clinical symptoms severity using two tools; the 18-item Brief Psychiatric Rating Scale (BPRS) and the Clinical Global Impression (CGI). BPRS is a seven-point Likert scale ranging from not present to extremely severe, it is used to assess the severity of psychopathology for participants with schizophrenia from the past week [18]. There are five symptom domains: affect (anxiety, depression, guilt, somatic); positive symptoms (thought content, conceptual disorganization, hallucinatory behaviour, grandiosity); negative symptoms (blunted affect, emotional withdrawal, motor retardation, disorientation); resistance (hostility, uncooperativeness, suspiciousness); and activation (excitement, tension, mannerisms–posturing) [19]. The total score of the BPRS showed very good reliability (the intraclass correlation *r* = 0.78, *p* < 0.001), and a good validity with the global estimate (a correlation = 0.66, *p* < 0.01) [20].

Besides, CGI is used for a brief assessment of a patient’s global functioning as average severity level across 7 days, for which its subscale for illness severity (CGI-S) was used to evaluate the symptom severity. This CGI-S is rated on a 7-point scale, using a range of responses from normal to the most severely ill. The tool has been shown to have good inter-rater reliability [21].

#### Well-being measurement

We evaluated the patient’s well-being, for which we used the Subjective Well-being under Neuroleptic treatment Scale (SWN). This is a self-reported measurement consisting of 20 items in six-point Likert scales, ranging from 1 to 6, which address the subjective experience of well-being under neuroleptic treatment in a patient with psychosis in five subscales: emotional regulation, self-control, mental functioning, social integration and physical functioning [22]. Total scores range from 20 to 120 indicating poor to excellent well-being, with a higher score referring to greater well-being [15]. A cut-off score ≥ 80 defined as ‘adequate well-being’ and those scores < 80 as ‘poor wellbeing’ [23]. Cronbach alpha was 0.95 for the total scores and 0.73–0.88 for the five subscales [14, 24]. SWN was forward translated into Thai by psychiatrists, then using back-translation to English by an independent, professional translator. A pre-test of the translated questionnaires assessing their practical usage and understanding was performed. The SWN Thai version showed good inter-rater reliability with Kappa of 0.88 (SD 0.24) [12].

### Data collection

On the day of the patient’s appointment, they were invited to participate in the study at an outpatient clinic using a convenient sampling method by a nurse at the clinic. The nurse was not a part of the researcher team. After obtaining informed consent, the patients completed the socio-demographic data and self-reported well-being: SWN, in the available room at the outpatient clinic – participant’s privacy was maintained at all times during data collection. The medical history and two symptom severity tools: BPRS and CGI-S were completed by their attending psychiatrists.

### Statistical analysis

Sample size calculation was based on a correlation coefficient (− 0.24) between symptom severity (Positive and Negative Syndrome Scale) and well-being (SWN emotional regulation subscale) from a previous study by Naber et al. [15] With 80% power and alpha of 0.05, a total of at least 134 participants was needed.

Data entry was performed using EpiData version 3.1. The R software version 3.4.1 (R Development Core Team, 2012) was used for data analyses. Categorical variables were presented as both frequencies and percentages, whereas continuous variables were presented as means with standard deviations or median with inter-quartile range. Group differences between adequate and poor well-being status were compared using Wilcoxon rank-sum test for non-parametric data. The patients’ SWN score was analyzed as both continuous and categorical variables (adequate or poor well-being). Correlations between the symptom severity and patients’ well-being SWN scores were analyzed using Pearson’s correlation coefficients. Strength of correlation (either positive or negative) was interpreted as follows: 0–0.10 as negligible correlation; 0.10–0.39 as weak correlation; 0.40–0.69 as moderate correlation; 0.70–0.89 as strong correlation; and 0.90–1.00 as very strong correlation [25]. The associations between ‘adequate’ or ‘poor’ well-being status and the symptom severity scores were analyzed using multivariate logistic regression analysis, adjusted for age, sex, education level, employment status, income, duration of illness, hospitalization, dose of antipsychotic drug, and adverse drug effect. A *p*-value of less than 0.05 was considered to indicate statistical significance.

## Results

### Patient characteristics and medical history

Amongst 150 patients with schizophrenia, mean age 42.7 years, nearly half of them were female. All participants were educated, and approximately 60% were employed. More participant characteristics and medical history details are presented in Table 1.

**Table 1:** Socio-demographic characteristics and medical history of patients with schizophrenia (*N* = 150)

Variables	Total N (%)
**Sex** (Female)	74 (49.3)
**Age** (years), Mean ± SD, min - max	42.7 ± 12.1; 18–70
≤ 35	45 (30.0%)
> 35–45	51 (34.0%)
> 45–55	29 (19.3%)
> 55	25 (16.7%)
**Highest Education**	
Primary school	20 (13.3)
High school or vocational education	76 (50.7)
Higher education	54 (36.0)
**Employment**	86 (57.3)
**Monthly income** (USD) ^a^	
No salary	33 (22.0)
≤ 157	46 (30.7)
> 157–313	34 (22.7
> 313	37 (24.7)
**Duration of illness** (years), Median (IQR); min - max	11 (5, 20); 8 days – 40 years
< 10	56 (37.3%)
11–20	54 (36.0%)
> 20	40 (26.7%)
**History of hospitalization** (time), Median (IQR); min - max	1 (1, 3), 1–10
Never	64 (42.7)
At least one time	46 (30.7)
More than one time	40 (26.7)
**Current antipsychotic treatment**	147 (98.0)
**Types of antipsychotic drug**	
Typical antipsychotic drug	63 (42.0)
Atypical antipsychotic drug	40 (26.7)
Both types of drug	44 (29.3)
Not currently received medication	3 (2.0%)
**Routes of medication**	
Oral	128 (85.3)
Intramuscular	2 (1.3)
Both	17 (11.3)
Not currently received medication	3 (2.0%)
**CPZ equivalent daily dose** (mg), Median (IQR); min-max	300 (150,500); 25–1004
≤ 100	26 (17.3%)
> 100–250	43 (28.7%)
> 250–500	51 (34.0%)
> 500	27 (18.0%)
Not currently received medication	3 (2.0%)
**Common medication side effects**	52 (34.7)
Drowsiness	18 (34.6)
Weight gain	10 (19.2)
Dizziness	6 (11.5)

*CPZ* Chlorpromazine, *IQR* Inter-Quartile Rank

^a^ 1USD = 31.90Baht (Source: Bank of Thailand (Foreign Exchange Rates as of 28 June 2021)). Retrieved from URL: https://www.bot.or.th/english/statistics/financialmarkets/exchangerate/_layouts/application/exchangerate/exchangerate.aspx)

### Correlations between symptom severity and patients’ well-being score

**Fig. 1:**
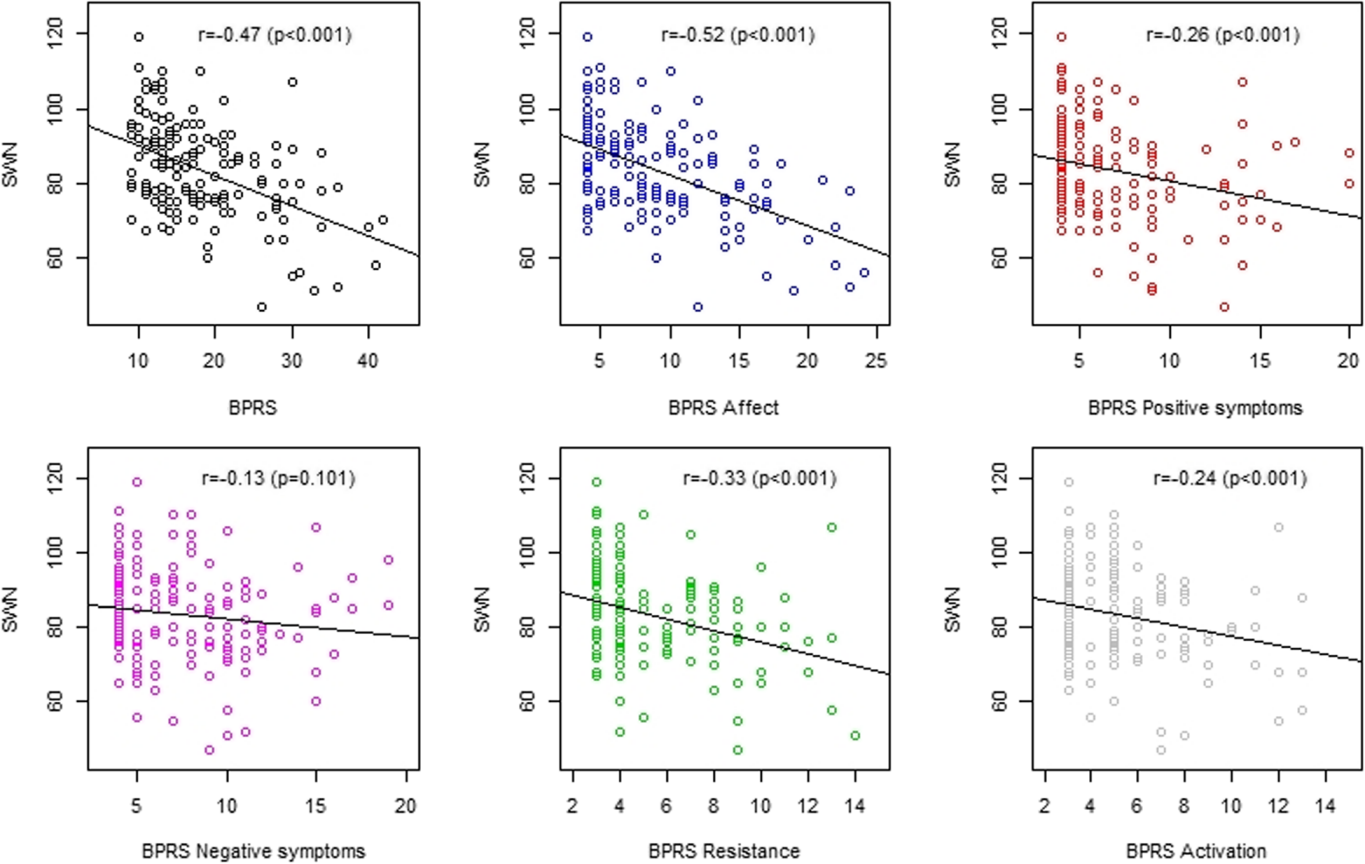
Correlation coefficients between BPRS with its domains and SWN score (*N* = 150)

BPRS showed the highest inverse correlation coefficient with SWN score (*r* = − 0.47; *p* < 0.001), which was considered as a moderate correlation. The CGI-S also showed significant negative correlations with the SWN score, however, the finding was defined as a weak correlation (*r* = − 0.21; *p* < 0.01). Considering the correlation analysis in BPRS domains and SWN, the BPRS Affect domain revealed the highest negative correlation (*r* = − 0.52; *p* < 0.001); followed by BPRS Resistance, Positive symptoms and Activation domain. (Fig. 1) There was no evidence for correlations between BPRS Negative symptoms domain and SWN score.

### Symptom severity with patients’ well-being status

Eighty-nine participants (59.3%) reported adequate well-being status. Considering symptom severity, the median score of BPRS was 18.1 (IQR 12, 21) and CGI-S was 2 (1, 3). Those patients with adequate well-being showed significantly lower BPRS score compared to others with poor well-being (median 14 (IQR 12, 20) vs 19 (14, 28); *p* < 0.001), while the CGI-S score showed no significant difference between adequate and poor well-being status (2 (1, 3) vs 2 (2, 3); *p* = 0.12). Table 2 describes more symptom domain scores of BPRS, patients with adequate well-being status significantly reported lower BPRS Affect (*p* < 0.001), Resistance (*p* = 0.01) and Positive symptoms scores (*p* = 0.02) than those with poor well-being.

**Table 2:** The characteristic of symptom severity domains of BPRS score categorized by ‘adequate’ or ‘poor’ well-being status of patients with schizophrenia (*N* = 150)

BPRS Symptom severity domains	Median (IQR), min - max	Adequate well-being	Poor well-being	***P***-value Ranksum test
Affect (4-item)	8 (5, 12), 4–24	6 (4, 10)	10 (7, 15)	< 0.001
Positive symptoms (4-item)	5 (4, 8), 4–20	5 (4, 7)	6 (4, 9)	0.02
Negative symptoms (4-item)	6.5 (4, 10), 4–19	6 (4, 9)	7 (5, 10)	0.16
Resistance (3-item)	4 (3, 7), 3–14	4 (3, 6)	4 (4, 8)	0.01
Activation (3-item)	4 (3, 6), 3–13	4 (3, 5)	5 (3, 8)	0.09

*BPRS* Brief Psychiatric Rating Scale

**Fig. 2:**
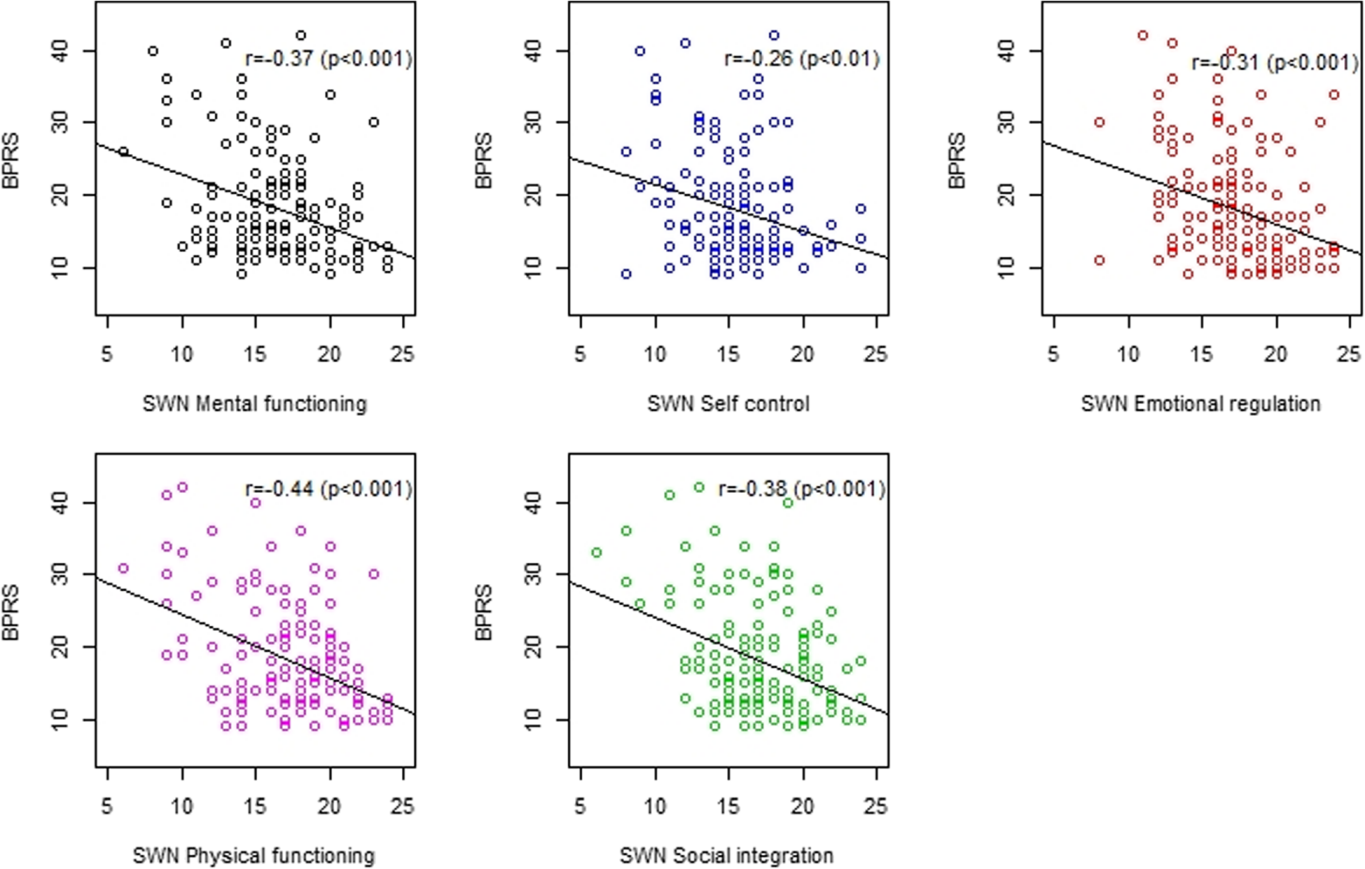
Correlation coefficients between SWN with its subscales and BPRS score (*N* = 150)

Due to BPRS showing better association than the CGI-S, we further explored whichever score of SWN subscale was associated with the BPRS score. We found that SWN Physical functioning showed the strongest correlation with the BPRS score (*r* = − 0.44, *p* < 0.001). Other SWN subscales were revealed to be significant, but were defined as weak, correlations, see Fig. 2.

### Association of symptom severity and patients’ well-being status

The univariate and multivariate logistic regression models of all variables are shown in Table 3. BPRS score and unemployment were significant in univariate analysis with OR 1.09 (95%CI 1.04–1.15; p < 0.001) and 2.17 (95%CI 1.11–4.26; *p* = 0.024), respectively. These two factors remained significantly associated with the patient’s well-being status in multivariate logistic regression, showing adjusted OR of BPRS score with 1.08 (95%CI 1.02–1.14; *p* = 0.006) and unemployment with 4.01 (95%CI 1.38–11.7; *p* = 0.011). There was no evidence of a relationship between CGI-S score and well-being status (OR 1.19 95%CI 0.88–1.61; *p* = 0.246).

**Table 3:** Univariate and multivariate logistic regression analyses of poor well-being status associated with BPRS score

Variables	Crude	***P***-value	Adjusted	***P***-value
	Odds Ratio (95%CI)	Wald test	Odds Ratio (95%CI)	Wald test
**BPRS**	*1.09 (1.04–1.15)*	*< 0.001*	*1.08 (1.02–1.14)*	*0.006*
**Sex** (ref: male)	0.96 (0.5–1.85)	0.896	1.10 (0.49, 2.46)	0.825
**Age** (ref: age ≤ 35)		0.199		0.296†
> 35–45	2.26 (0.98–5.22)	0.056	2.73 (0.91, 8.19)	0.073
> 45–55	1.05 (0.39–2.82)	0.919	1.35 (0.40, 4.51)	0.631
> 55	1.20 (0.43–3.37)	0.729	1.56 (0.37, 6.52)	0.542
**Education** (ref: primary school)		0.396		0.523†
High/vocational school	0.52 (0.18–1.47)	0.219	0.50 (0.15, 1.67)	0.259
Higher degree	0.48 (0.16–1.43)	0.19	0.57 (0.15, 2.20)	0.415
**Unemployment** (ref. employment)	*2.17 (1.11–4.26)*	*0.024*	*4.01 (1.38, 11.7)*†	*0.011*
**Monthly income** (USD) ^§^ (ref: no salary)		0.438		0.250†
≤ 157	1.18 (0.47–2.94)	0.723	2.86 (0.87, 9.44)	0.085
> 157–313	1.07 (0.41–2.82)	0.889	3.13 (0.81, 12.07)	0.098
> 313	0.57 (0.21–1.54)	0.27	1.80 (0.43, 7.50)	0.417
**Duration of illness** (ref: < 10 years)		0.622		0.827†
11–20	1.39 (0.64–3.00)	0.404	1.18 (0.47, 3.00)	0.726
> 20	0.97 (0.42–2.25)	0.946	0.86 (0.26, 2.88)	0.813
**History of hospitalization** (ref: never)		0.528		0.142†
At least one time	1.07 (0.49–2.31)	0.87	1.40 (0.55, 3.59)	0.483
More than one time	0.67 (0.29–1.53)	0.339	0.44 (0.15, 1.27)	0.13
**CPZ equivalent daily dose** (ref: ≤ 100 mg)		0.58		0.801†
> 100–250	1.47 (0.52–4.13)	0.464	1.25 (0.37, 4.22)	0.719
> 250–500	2.00 (0.74–5.43)	0.173	1.75 (0.53, 5.79)	0.36
> 500	1.55 (0.5–4.80)	0.45	1.23 (0.33, 4.62)	0.755
**Adverse drug effects** (ref: none)	0.90 (0.45–1.81)	0.774	0.94 (0.40, 2.20)	0.889

*BPRS* Brief Psychiatric Rating Scale, *CPZ* Chlorpromazine

† *P*-value Likelihood-ratio test

^§^ 1USD = 31.90Baht (Source: Bank of Thailand (Foreign Exchange Rates as of 28 June 2021)). Retrieved from URL: https://www.bot.or.th/english/statistics/financialmarkets/exchangerate/_layouts/application/exchangerate/exchangerate.aspx)

## Discussion

The present study aimed to determine the associations between symptom severity and patients’ well-being in schizophrenia. As hypothesized, the inverse relationship was found: the higher the symptom severity scores of patients with schizophrenia, the lower the patients’ well-being. BPRS presented a greater correlation with well-being than the CGI-S score. BPRS Affect domain was more dominant than other BPRS domains. Two-thirds of patients with schizophrenia in our study had adequate well-being status. The BPRS score and being unemployed significantly predicted patients’ well-being status in multivariate regression analyses.

Consistent with the literature, this study found that patients who reported higher symptom severity had lower subjective well-being, [14–16] with BPRS the most negatively correlated with well-being score. From multivariate logistic regression analyses, the BPRS score consistently revealed a significant association with poor well-being status. This study confirms that symptom severity is negatively associated with patient’s well-being amongst low- and middle-income countries. When considering symptom domains, BPRS Affect was the most associated with well-being score since its correlation effect was considered moderate. These relationships may partly be explained by the fact that emotional disturbances such as depression and anxiety were found to be significant variables associated with patients’ well-being, [10, 13, 26] which commonly occur through the course of illness [8]. Other BPRS domains: Positive symptoms, Resistance and Activation revealed significant, but weak, correlation with well-being score; only BPRS Negative symptoms showed no statistical significance, in contrast to earlier findings that negative symptoms are associated with patients’ well-being [27]. This inconsistency may be due to the psychometric properties of BPRS measuring fewer dimensions of negative symptoms [28]; or the deficit of motivation, goal-directed activities or experiencing well-being amongst patients with schizophrenia [29]. This may lead them to minimize complaints or under-report their subjective concerns.

Our analyses also tested the association of BPRS score with SWN subscales. Our study found the SWN Physical functioning had the strongest correlation with the BPRS score. This might be because patients’ concern may be attributed more to a physical condition that is easily observed by themselves. Furthermore, SWN was objectively developed to assess patient’s well-being experiences affected by antipsychotic medications [15] and their side effects (e.g. akathisia, extrapyramidal symptoms) [13, 30]. Other SWN subscales were significant, albeit weakly, correlated to the BPRS score.

Unlike BPRS, the CGI-S did not show association with well-being status in a logistic regression analysis. Such observation could be due to the fact that CGI-S utilized only one question item, with only 7 levels of response, to evaluate symptom severity, and that may lead to less variability of the data (less data granularity) compared to the BPRS.

Additionally, the multivariate regression model demonstrated that being unemployed was also significantly associated with poor well-being status, independent of symptom severity. This accords with previous research, which showed that employment could help assess the status of patients’ well-being [31].

To our knowledge, this is the first study focusing on the associations of several domains of symptom severity with well-being amongst patients with schizophrenia amongst developing countries. We also observed various clinical characteristics for data analysis; not only symptom severity but also clinical factors (e.g. medication profiles, medication side effects) and other factors that may contribute to the patient’s well-being (e.g. education, employment).

This study was limited firstly by the study design being cross-sectional in nature and therefore could not imply a causal relationship between symptom severity and well-being. Secondly, the BPRS used in this study measured fewer dimensions of negative symptoms; comprehensive assessment of the negative symptoms should be further studied. Thirdly, the use of convenience sampling method might potentially lead to selection bias and might not be fully representative of study participants. Lastly, we did not include other psychotropic medications (e.g. antidepressants, mood stabilizers), objective measurement (e.g. functional and vocational outcomes), or factors about supportive system and family stress in this study, as these factors may affect symptoms, functions, and well-being in patients with schizophrenia [32].

An implication of this study is that clinicians need to optimize treatment to reduce symptoms as this will improve the well-being of patients with schizophrenia. Our findings showed that severity of symptoms as measured by BPRS is associated with well-being among patients with schizophrenia. The incorporation of BPRS into routine clinical practice could serve as an adjunct to physician clinical evaluation of patients’ symptoms. The symptom checklist of BPRS may help detect symptoms that patients may underreport, or physicians may be less concerned about in a busy clinical setting. By performing this double assessment, we could monitor symptom severity, and identify specific symptoms, which may help improve patients’ well-being. Although we emphasized the usefulness of utilizing objective measurement, the physician’s assessment will always be needed because it is swift, pleasant, professional, and highly regarded by society.

## Conclusions

In summary, inverse relationships were found between symptom severity and well-being score, with the highest coefficient between BPRS and the patient’s well-being. BPRS Affect was the most significantly associated with well-being score. Despite no active symptoms, just two-thirds of patients achieved adequate well-being status. The use of the BPRS score and being unemployed were significantly associated with predicting poor well-being status. Further study should assess more dimensions of negative symptoms associated with well-being in schizophrenia.

## Data Availability

The datasets generated and/or analysed during the current study are not publicly available due the data set containing sensitive personal health information but are available from the corresponding author on reasonable request.

## Abbreviations

BPRS: 18-item Brief Psychiatric Rating Scale
CGI: Clinical Global Impression
CPZ: ChlorPromaZine
SWN: Subjective Well-being under Neuroleptic treatment scale
